# Evolution of Immune Systems From Viruses and Transposable Elements

**DOI:** 10.3389/fmicb.2019.00051

**Published:** 2019-01-29

**Authors:** Felix Broecker, Karin Moelling

**Affiliations:** ^1^Department of Microbiology, Icahn School of Medicine at Mount Sinai, New York, NY, United States; ^2^Institute of Medical Microbiology, University of Zurich, Zurich, Switzerland; ^3^Max Planck Institute for Molecular Genetics, Berlin, Germany

**Keywords:** transposable elements, mobile genetic elements, viruses, superinfection exclusion, immune system, CRISPR-Cas, antibodies, RNase H

## Abstract

Virus-derived sequences and transposable elements constitute a substantial portion of many cellular genomes. Recent insights reveal the intimate evolutionary relationship between these sequences and various cellular immune pathways. At the most basic level, superinfection exclusion may be considered a prototypical virus-mediated immune system that has been described in both prokaryotes and eukaryotes. More complex immune mechanisms fully or partially derived from mobile genetic elements include CRISPR-Cas of prokaryotes and the RAG1/2 system of vertebrates, which provide immunological memory of foreign genetic elements and generate antibody and T cell receptor diversity, respectively. In this review, we summarize the current knowledge on the contribution of mobile genetic elements to the evolution of cellular immune pathways. A picture is emerging in which the various cellular immune systems originate from and are spread by viruses and transposable elements. Immune systems likely evolved from simple superinfection exclusion to highly complex defense strategies.

## Introduction

**FIGURE 1:**
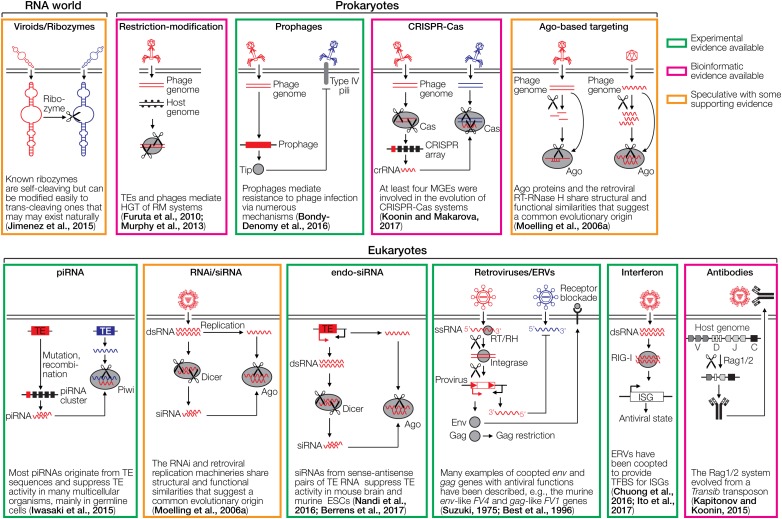
Cartoons depicting various defense systems. The systems are color-coded based on the level of support in green (experimental evidence available), magenta (bioinformatic evidence available), and orange (speculative with some supporting evidence). DNA/RNA cleaving is indicated with scissors. RNA is depicted as wavy lines or with secondary hairpin-loop structures. A ribozyme cleaving another ribozyme is a hypothetical early immune system that does not require DNA or proteins. The ribozyme may be part of a viroid-like selfish RNA. Restriction-modification systems distinguish between foreign and self DNA by methylating target sequences of restriction endonucleases. Prophages can mediate superinfection exclusion, exemplified by expression of the Tip protein that reduces bacterial surface expression of type IV pili required for infection by various phages. CRISPR-Cas acts by incorporating small genomic fragments from phages into CRISPR arrays in the prokaryotic genome. The transcribed spacers are then used by another Cas member to cleave sequence-homologous phage genomes. PIWI-associated RNAs (piRNAs) are small RNAs complementary to transposable elements (TEs) that are encoded in piRNA clusters (Iwasaki et al., 2015). piRNAs associate with a PIWI nuclease to cleave complementary TE transcripts. RNA interference (RNAi) is initiated by dsRNA which is fragmented by Dicer to siRNAs. These siRNAs are loaded into the RNA-induced silencing complex to cleave complementary RNAs using Ago nucleases. A variation of RNAi is the endo-siRNA pathway, in which dsRNA is generated from TEs that are transcribed in both orientations, for instance, if the TE is located in an intron in opposite orientation to the encompassing gene. Endogenous retroviruses (ERVs) can mediate restriction of ERVs and exogenous retroviruses through various mechanisms, including receptor blockade by captured Env proteins, Gag-mediated restriction and antisense RNA mechanisms. The interferon system recognizes dsRNA or other pathogen-associated molecular patterns, which leads to upregulation of antiviral interferon-stimulated genes (ISGs). The antibody system involves diversification through light and heavy chain recombination, which is mediated by the Rag recombinases. This enables the detection of diverse pathogens. Note that the references provided in the figure are not comprehensive. Please refer to the text for more details and additional references.

Cellular organisms have co-evolved with various mobile genetic elements (MGEs), including transposable elements (TEs), retroelements and viruses, many of which can integrate into the host DNA. MGEs constitute ∼50% of mammalian genomes, >70% of some plant genomes and up to 30% of bacterial genomes (Koonin and Krupovic, 2015). The evolutionary interplay between MGEs and their hosts has generated a plethora of cellular defense mechanisms and counter-measures. Notably, many immune systems, or parts thereof, including the prokaryotic CRISPR-Cas mechanism and antibody/T cell receptor (TCR) diversification by V(D)J recombination in vertebrates have been recruited from viruses or other MGEs. Here, we summarize the current knowledge on the evolution of diverse immune systems of prokaryotes and eukaryotes, highlighting a general scenario for the origin of cellular defense systems from MGEs. A non-exhaustive overview of different cellular immune systems is presented in Figure 1.

## Viruses Against Viruses: Superinfection Exclusion as a Mechanism of Antiviral Immunity

Superinfection exclusion (SIEx) is the ability of a preexisting viral infection to restrict secondary infections, often by the same or a closely related virus. SIEx was first observed in tobacco plants that, when pre-infected with a mild variant of *Tobacco mosaic virus* (TMV), were protected against a virulent TMV strain (McKinney, 1929). SIEx was later found to be common for many other systems, including viruses of bacteria, animals, humans, and plants (Moelling et al., 2017). The cellular organism benefits from SIEx if a preexisting infection with a non-pathogenic or mildly pathogenic virus protects against detrimental viruses. Thus, SIEx can be regarded as a simple adaptive immune system, which is inheritable if the first virus integrates into the cellular genome or is transmitted to the progeny by other means. One recent experimentally verified example is Mavirus, a virophage that integrates into the genome of *Cafeteria roenbergensis* and protects the flagellate organism from infection with a deadly virus (Fischer and Hackl, 2016). This example is further described below.

An evolutionarily early immune system may have been constituted by viroids or viroid-like RNAs. Viroids are virus-related, protein-free infectious agents consisting of highly structured, circular non-coding RNA that can be catalytically active through ribozyme activity (Flores et al., 2014). They may be remnants of the ancient RNA world thought to have existed before the evolution of DNA or proteins (Diener, 1989; Flores et al., 2014). However, the fact that extant viroids have so far only been identified in plants (with the notable exception of hepatitis delta virus, a derivative of a viroid with a short insert of protein-coding capacity) suggests their appearance after the last universal cellular ancestor (Koonin and Dolja, 2014). Regardless, viroids likely recapitulate principal features of selfish elements of the ancient RNA world. In plants, SIEx has been described between mild and severe strains of the same viroid as well as between different viroids (Kovalskaya and Hammond, 2014). The mechanisms of SIEx in plants may include RNA interference (RNAi), with siRNAs produced by Dicer from the first infecting viroid acting against the superinfecting one. It remains unclear, however, how the first viroid escapes RNAi; it may associate with protecting host factors or its localization in the nucleus or chloroplasts protects from RNAi, which mainly acts in the cytoplasm (Kovalskaya and Hammond, 2014). It seems likely that SIEx existed before the evolution of complex viruses or cellular immune systems such as RNAi. In the ancient RNA world, a simple RNA-based immune system could have been constituted of a ribozyme/viroid that prevents superinfection with another one *via* ribozymatic cleavage *in trans* (Figure 1). Although known natural ribozymes/viroids are generally self-cleaving, they can be modified relatively easily to yield *trans*-cleaving derivatives (Jimenez et al., 2015), suggesting that *trans*-cleaving ribozymes may have existed or may still exist naturally.

## Integrated Viral Sequences Act as Inheritable Immunity in Prokaryotes

Insertion of viral genomes, or parts thereof, into host genomes is at the origin of many immune systems (Moelling et al., 2017). Integration of prophages into bacterial genomes is often associated with a fitness cost to the host (Iranzo et al., 2017), however, prophages can mediate resistance to infection by exogenous bacteriophages (phages) through various mechanisms. For example, the prophage-encoded Tip protein inhibits formation of type IV pili on the surface of *Pseudomonas aeruginosa* (Chung et al., 2014). Since these pili are common phage receptors, Tip expression mediates SIEx to various phages (Bondy-Denomy et al., 2016). Interestingly, prophage-mediated alteration of type IV pili function has little or no fitness cost to the host. In *P. aeruginosa*, three prophages are sufficient to mediate resistance against at least 30 different phages. Various other mechanisms of prophage-mediated protection against exogenous phages have been reported in multiple bacterial species, which include cell surface alterations, receptor blockade and transcriptional repression (Bondy-Denomy and Davidson, 2014).

CRISPR-Cas provides another prokaryotic adaptive immune system. Here, a fragment of DNA (or reverse-transcribed RNA) of an infecting phage or other foreign genetic elements is integrated as spacer into a CRISPR array in the host genome (Hille et al., 2018). Thereby, spacers act as immunological memory for long-term protection of the cell and future generations. The transcribed CRISPR array RNA (pre-crRNA) is processed into smaller crRNAs that guide sequence-specific cleavage of homologous invading nucleic acids by Cas effector nucleases. A common feature of all Type II and Type V effector nucleases, Cas9 and Cas12a, respectively, is a ribonuclease H (RNase H)-like RuvC domain that cleaves the non-complementary DNA strand, whereas an HNH nuclease (Cas9) or a NUC domain (Cas12a) cleaves the complementary DNA strand of the target dsDNA (Makarova et al., 2017). Other Cas effectors utilize different nuclease domains, such as histidine-aspartate nucleases. CRISPR arrays are found in about half of bacterial and nearly 90% of archaeal genomes (Hille et al., 2018).

At least four different MGEs were involved in the evolution of CRISPR-Cas systems (Koonin and Makarova, 2017). First, the adaptation module of all CRISPR-Cas systems, responsible for spacer acquisition, originated from casposons (a fusion word between Cas and transposons), TEs that utilize Cas1 nuclease for DNA integration (Krupovic et al., 2014). Second, the Cas2 nuclease, which could have been present also in the casposon, as well as HEPN family RNases found in several other Cas proteins likely originate from toxin-antitoxin (TA) modules (Koonin and Krupovic, 2015). Although TA modules do not encode their own mobility genes, they can be regarded as MGEs, as they are typically transferred by plasmids (Koonin and Makarova, 2017). Third, many Type III CRISPR-Cas systems recruited the reverse transcriptase (RT) from a mobile group II intron, which allows for spacer acquisition from invading RNA. Fourth, the RuvC domains of Type II and Type V systems were likely derived from TnpB nucleases of DNA transposons. A complete, functional CRISPR-Cas system encoded by a phage has been reported (Seed et al., 2013), suggesting that phages may also serve as vehicles for horizontal gene transfer (HGT) of this kind of immune system. Both prophage-mediated SIEx and CRISPR-Cas can be regarded as adaptive prokaryotic immune systems that generate immunological memory to protect the cell and future generations against viral infections. CRISPR-Cas acquired specific immunity can be transmitted across thousands of microbial generations (Weinberger et al., 2012).

## Bacterial Immune Systems Co-Localize With viral and Transposable Element Sequences

Restriction-modification (RM) systems consist of two components; a restriction endonuclease that cleaves invading double-stranded DNA (e.g., of phage origin) by recognizing a short DNA motif and a methylase that masks that motif on the prokaryote’s genome by introducing methyl groups to prevent destruction of its own DNA. Since the recognition motifs are usually short and thereby likely to be present on the majority of invading DNA molecules, RM systems can be regarded as a prokaryotic innate immune system. The various restriction endonucleases of RM systems likely evolved from one or a few common ancestor(s) (Jeltsch et al., 1995) and became widespread *via* HGT (Jeltsch and Pingoud, 1996). RM systems are encoded by about 90% of prokaryotes (Murphy et al., 2013). Various phages have been shown to be able to mediate HGT of RM genes, indicating that phages are common vectors for these immune systems (Murphy et al., 2013). RM genes frequently co-localize with viral and TE sequences such as integrases and transposases and in some cases are flanked by inverted repeats and target site duplications, hallmarks of TEs (Naderer et al., 2002; Furuta et al., 2010; Makarova et al., 2011; Takahashi et al., 2011). TEs carrying functional RM systems have been identified (Khan et al., 2010), raising the possibility that these defense systems evolutionarily originate from TEs. Some restriction endonucleases can also trigger programmed cell death of bacteria (Nagamalleswari et al., 2017). This phenomenon of ‘bacterial apoptosis’ has been described as a mechanism that occurs upon phage infection to limit spread of the virus, reminiscent of eukaryotic apoptosis triggered by viral infection (Chopin et al., 2005).

A number of additional prokaryotic innate anti-phage systems have recently been identified (Koonin, 2018). These include prokaryotic Ago proteins that cleave invading DNA or RNA with RNase H-like nuclease domains (Swarts et al., 2014), BREX, which blocks phage replication and methylates bacterial DNA, enabling BREX to differentiate between host and phage genomes (Goldfarb et al., 2015) and DISARM, which also methylates host DNA and restricts invading dsDNA phages (Ofir et al., 2017). In addition, a number of defense systems were recently identified by a systematic search for genes clustering with defense islands, regions involved in defense processes that are widely abundant in prokaryotic genomes (Makarova et al., 2011; Doron et al., 2018). Ten of the novel defense systems were verified experimentally *in vitro* either in *Escherichia coli* or *Bacillus subtilis* that became resistant to a panel of phages upon introduction of the defense system. Interestingly, TE sequences are enriched in defense islands (Doron et al., 2018). The majority of prokaryotic TEs encode DDE superfamily transposases with an RNase H fold (named after two aspartate and one glutamate residue that form a catalytic triad) that mediate mobility *via* a cut-and-paste mechanism (Koonin and Krupovic, 2015). It remains unknown if these TEs serve a functional role, or whether their accumulation in defense island is simply less deleterious compared to other genomic loci. It is tempting to speculate, however, that some of the defense island-associated TE sequences, especially the RNase H-like transposases, may have been captured by the host to fulfill defense functions, or that they have contributed to the spread of immune systems.

## Protection From Retroviral Infection by Endogenized *env* Genes

**FIGURE 2:**
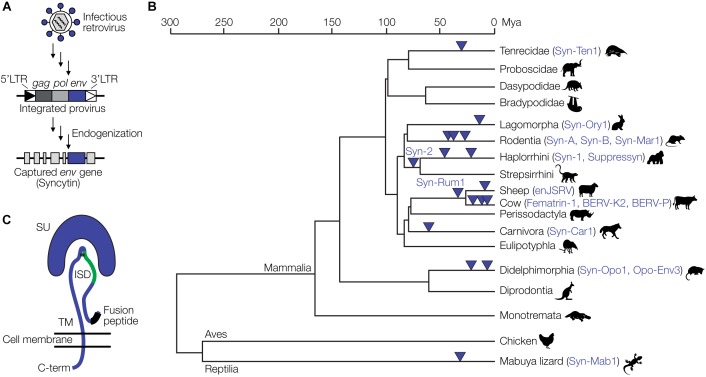
Capture of retroviral genes for placental development. **(A)** Schematic of endogenization and capture of a syncytin gene. A retrovirus infecting a germline cell integrates into the host genome as a provirus with characteristic features, the 5′LTR, *gag, pol* and *env* genes, and the 3′LTR. Over time, in a process termed endogenization, most of the provirus acquires deleterious mutations. In this example, the *env* gene retains an intact open reading frame and is captured by the host to fulfill functions during placental development. **(B)** Evolution of placental species and capture of ERV-derived *env* genes. The phylogenetic tree is based on previously published data (Cornelis et al., 2017; Imakawa and Nakagawa, 2017). **(C)** Structural representation of the retroviral Env protein with the surface (SU), transmembrane (TM), and immunosuppressive domain (ISD) subunits, as well as the fusion peptide. Panel **(C)** has been modified from Lavialle et al. (2013).

Eukaryotic genomes harbor large amounts of endogenous retrovirus (ERV) sequences, which are remnants of retroviral infections of ancestral germline cells. The human and mouse genomes, for instance, contain about 8% and 10% ERV sequences, respectively (Gifford and Tristem, 2003; Broecker et al., 2016). Among the best studied examples of retroviral genes that have been captured by mammalian (and some reptilian) hosts are the syncytins (Lavialle et al., 2013). Syncytins originate from *env* genes of endogenized proviruses. Full-length proviruses harbor the three retroviral genes, *gag, pol* and *env*, flanked by two LTRs. ERVs not subject to any selective pressure are inactivated by mutation to various degrees over time (Broecker et al., 2016). In rare occasions, however, certain proviral genes have been conserved over millions of years of evolution (Figure 2A), suggesting a selective advantage of that gene to the host. Retroviral *env* genes have been repeatedly captured from different proviruses at least 17 times during evolution and as syncytins or related genes exert critical physiological functions in the placental development of various mammalian and viviparous lizard species (Figure 2B) (Cornelis et al., 2017; Imakawa and Nakagawa, 2017).

Syncytins also likely contribute to maternal immune tolerance toward the fetus *via* the immunosuppressive domain (ISD) (Figure 2C). The ISD has been demonstrated to exert various immunosuppressive functions *in vitro* and *in vivo*, including an inhibition of the activity of lymphocytes, natural killer cells, monocytes and macrophages as well as the downregulation of pro-inflammatory cytokines (Cianciolo et al., 1985; Haraguchi et al., 1995, 1997, 2008). An *env*-derived syncytin gene has recently been identified in viviparous lizards that possess a mammalian-like placenta (Cornelis et al., 2017). Thus, syncytin capture is not restricted to mammals and likely a hallmark of placental evolution in general.

In addition to syncytins and related genes, other retroviral *env* genes have been captured for anti-retroviral functions. The first described example, the *Friend virus susceptibility 4* (*Fv4*) gene, confers resistance to murine leukemia viruses (MuLVs) in mice (Suzuki, 1975). *FV4* is a truncated MuLV-related provirus containing the 3′ portion of *pol*, the entire *env* gene and the 3′LTR (Ikeda et al., 1985). Binding of the *env*-encoded protein to the cellular receptor used by MuLV prevents infection by the exogenous retrovirus, a process termed receptor blockade. Another captured *env* gene in mice, *resistance to MCF* (*Rmcf*), mediates resistance to mink cell focus-inducing (MCF) viruses and MuLVs, likely also *via* receptor blockade (Hartley et al., 1983; Brightman et al., 1991; Jung et al., 2002).

Jaagsiekte sheep retrovirus (JSRV) co-exists both as exogenous and endogenous form (Armezzani et al., 2014). JSRV is an example of recent or ongoing endogenization, with the youngest identified endogenous elements (enJSRV) having integrated about 200 years ago. The sheep genome harbors at least 27 enJSRV sequences, 16 of which with intact *env* genes. enJSRV *env* is expressed in the ovine placenta and knockdown with antisense oligonucleotides has been shown to cause defects in trophoblast differentiation and pregnancy loss (Dunlap et al., 2006). This demonstrates that even recently endogenized, mostly intact *env* genes can exert syncytin-like functions. In addition, enJSRV *env* expression has been shown to block exogenous JSRV *via* receptor blockade (Spencer et al., 2003).

In cats, the *refrex-1* gene mediates resistance to feline leukemia virus-D (FeLV-D) (Ito et al., 2013). *Refrex-1* is a truncated retroviral *env* gene, such that the protein contains the signal peptide (SP) and the N-terminus of the surface unit (SU) that is a putative receptor-binding domain, but lacks the C-terminus of SU and the transmembrane (TM) domain due to a premature stop codon (Figure 2C).

In human cells, it has been recently shown that the Env protein encoded by a HERV-K(HML-2) provirus interferes with HIV-1 production *in vitro* through an unknown mechanism (Terry et al., 2017). The provirus, HERV-K108, encodes full-length Env with four mutations compared to the consensus, ancestral HERV-K(HML-2) protein. These mutations appear to be required for inhibiting HIV-1. Interestingly, HERV-K(HML-2) expression in T cells is increased upon HIV-1 infection (Contreras-Galindo et al., 2007; Gonzalez-Hernandez et al., 2012). It is therefore tempting to speculate that inducible HERV-K(HML-2) proviruses have been evolutionarily conserved to express Env (and Gag, see below) to protect against exogenous retroviruses such as HIV. Another example of an evolutionarily conserved *env* gene with antiviral function in human cells is HERV-T *env* (Blanco-Melo et al., 2017). Expression of this gene mediates resistance to a reconstructed infectious HERV-T virus (the virus is extinct) by receptor blockade. Interestingly, the consensus *env* of the resurrected virus, but not the single endogenized *env* gene could be used by the virus for successful infection. This indicates that the *env* gene has been modified evolutionarily to bind to the viral receptor while losing its ability to constitute infectious virions. In addition, *Suppressyn*, a truncated *env* gene from a HERV-F element with a known role in placental development (see above), has been suggested to serve as restriction factor for exogenous retroviruses (Malfavon-Borja and Feschotte, 2015).

## Protection From Retroviral Infection by Endogenized *gag* Genes

Another retroviral gene that has been frequently captured by mammalian hosts is *gag*. The best studied *gag*-derived restriction factor is the mouse gene *Friend virus susceptibility 1* (*Fv1*) (Best et al., 1996). *Fv1* inhibits MuLV at a stage post-entry but before integration of the provirus, with the exact mechanism still unknown. The Fv1 protein has been shown to interact with the retroviral capsid protein (CA) in the preintegration complex of MuLV (Best et al., 1996). *Fv1* originates from a MERV-L *gag* gene that has little sequence homology with that of MuLV, implying that Fv1 and its target share structural properties despite few sequence similarities. In sheep, enJSRV expressed Gag has been shown to inhibit virion formation of exogenous JSRV, however, in contrast to Fv1, at a late stage during viral assembly (Palmarini et al., 2004). In human cells, the HERV-K(HML-2) CA protein inhibits release of HIV-1 and reduces infectivity of progeny HIV-1 virions (Monde et al., 2017).

## Other Antiviral Effects Mediated by Endogenous Retroviruses

**FIGURE 3:**
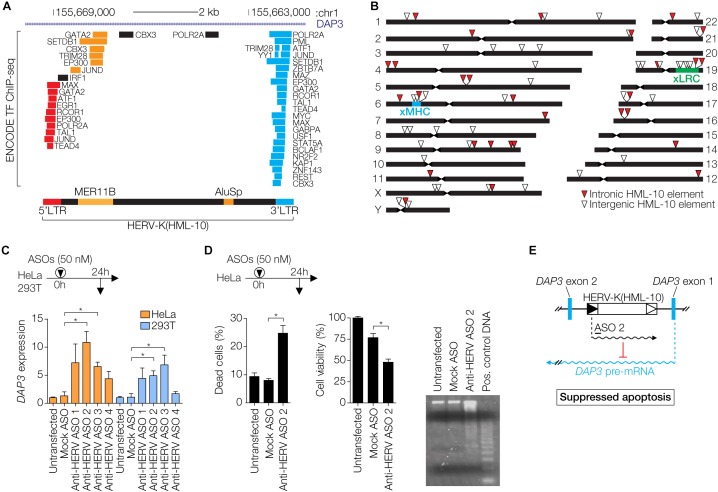
Regulatory functions of HERV-K(HML-10) elements. **(A)** Genomic region of a HERV-K(HML-10) provirus in the first intron of the *DAP3* gene. Repeat elements are according to RepeatMasker (Smit et al., 2013–2015) annotation. Two retroelements, *MER11B* and *AluSp*, are integrated into the provirus. Occupied transcription factor binding sites, as determined by ENCODE (Davis et al., 2018), are indicated and color-coded according to localization within the HERV-K(HML-10) provirus. Image modified from the UCSC Genome Browser (Kent et al., 2002) with the hg19 release of the human genome. **(B)** Chromosomal distribution of HERV-K(HML-10) elements in the human genome. The locations of the extended major histocompatibility complex (xMHC) and the extended leukocyte receptor complex (xLRC) are indicated. **(C)** Cells were transfected with indicated antisense oligonucleotides (ASOs). Anti-HERV ASOs target a HERV-K(HML-10) derived regulatory transcript, Mock ASO has an unrelated sequence. At 24 h after transfection, *DAP3* mRNA expression levels were determined by qRT-PCR and normalized to *GAPDH* levels. Bars show mean ± SEM of three experiments, untransfected cells were set to 1. ^∗^*P* ≤ 0.05, Student’s *t*-test against Mock. **(D)** HeLa cells were transfected with the indicated ASOs. After 48 h, Trypan Blue exclusion as indicator of dead cells (left) and MTS cell viability assays (center) were performed. The right subpanel shows genomic DNA of these cells prepared with the Apoptotic DNA Ladder Kit (Roche) and analyzed on an ethidium bromide stained agarose gel. The positive control DNA is from apoptotic U937 cells provided with the kit. Bars show mean ± SEM of three experiments in duplicates. ^∗^*P* ≤ 0.05, Student’s *t*-test. **(E)** Model of regulation of *DAP3* expression by the HERV-K(HML-10) primed regulatory transcript. The approximate location of ASO 2 is indicated. This figure shows data modified from Broecker et al. (2016).

In addition to Env- and Gag-mediated restriction, more indirect mechanisms of antiviral protection by ERVs have been described. In human cells, the HERV-K Rec protein expressed during early embryogenesis activates innate immune responses by inducing expression of the *IFITM1* gene, which protects the cell from viral infection (Grow et al., 2015). Moreover, ERVs have introduced and amplified interferon (IFN)-inducible enhancers within eukaryotic genomes and provide transcription factor binding sites (TFBS) that are enriched in proximity to genes involved in immune pathways (Chuong et al., 2016; Ito et al., 2017). This suggests that ERV sequences have been specifically adopted by host cells to modulate IFN responses, a major branch of the antiviral immune defense. An example in the human genome is the HERV-K(HML-10) family recently described by us and others (Broecker et al., 2016; Grandi et al., 2017). TFBS within the LTRs are frequently occupied, as determined by the ENCODE project (Davis et al., 2018), especially in the human myelogenous leukemia cell line K562 (Figure 3A). HERV-K(HML-10) elements appear to be enriched in loci involved in immunity such as the extended major histocompatibility complex (xMHC) and the extended leukocyte receptor complex (xLRC) (Barrow and Trowsdale, 2008; Horton et al., 2004) (Figure 3B). The data suggests that HML-10 has been captured by the host for the regulation of immune-related genes.

We have recently described a HERV-K(HML-10) provirus that regulates *DAP3* gene expression *in vitro* through an antisense mechanism, likely *via* a long non-coding RNA (Figure 3C) (Broecker et al., 2016). Inactivating the HERV-derived RNA by antisense oligonucleotides was sufficient to induce apoptosis *in vitro* (Figure 3D), demonstrating that HERV-mediated antisense regulation can directly influence the cellular phenotype (Figure 3E). In addition, there is evidence for HERV-mediated gene regulation in humans *in vivo*. The complement component *C4* gene in the xMHC exists in two variants, one with a transcriptionally active intron-located HERV-K(HML-10) provirus, and one without it (Schneider et al., 2001; Yang et al., 2003; Mack et al., 2004; Broecker et al., 2016). The presence of the provirus correlates with lower C4 protein serum concentrations, indicating that the HERV regulates *C4* expression, perhaps *via* an antisense mechanism (Yang et al., 2003). The fact that HERV-derived RNA can regulate expression of cellular genes also suggests that HERV antisense transcripts arising from intronic proviruses could suppress mRNA of exogenous retroviruses. In support of this, HERV antisense transcripts arising from intron-located proviruses have been shown to suppress complementary retroviral transcripts *in trans* (Schneider et al., 2001; Mack et al., 2004). Recombination between exogenous retroviruses and ERVs is another restriction mechanism, which may occur when defective ERV transcripts are co-packaged with the intact retroviral RNA into the same virion, or at the level of proviral DNA during meiosis (Löber et al., 2018).

## Endogenization in Real Time: Koala Retrovirus

An ongoing retroviral endogenization occurs in koalas. Koala retrovirus (KoRV) co-exists as both exogenous and endogenous form (Stoye, 2006; Tarlinton et al., 2006). High KoRV viral loads are associated with fatal lymphoid neoplasia. Since integration sites and copy numbers of proviruses are heterogeneous among individuals, and some koala populations isolated from mainland Australia since around the year 1900 are free of KoRV, the virus has likely entered the koala genome only about 100 years ago and is still undergoing endogenization. KoRV endogenization is possibly associated with resistance of koalas against the exogenous virus (Colson et al., 2015). To date it is unclear if Env or Gag-mediated restriction mechanisms may protect from exogenous KoRV. However, many endogenous proviruses have *gag* and *env* genes with complete open reading frames (ORFs), usually with point mutations (Oliveira et al., 2007). The mutated KoRV genes, when incorporated into retroviral vectors based on the closely related gibbon ape leukemia virus (GALV) that uses the same entry receptor as KoRV substantially reduced infectivity compared to the GALV *gag* and *env* genes. This suggests that KoRV endogenization is associated with mutations that render the proviruses incapable of producing highly infectious viruses. Yet, preservation of intact *gag* and *env* ORFs may indicate a functional importance, perhaps as restriction factors against exogenous KoRV. Moreover, some koala populations such as those located in Southern Australia (SA) are relatively resistant to KoRV induced disease and usually have low viral loads (Tarlinton et al., 2017). In contrast, koalas in northern Australia such as Queensland suffer from higher viremia and disease burden, and active KoRV infection is more prevalent. Of note, both populations have endogenous KoRV proviruses which, however, differ in their RNA expression patterns. While koalas from Queensland express mainly full-length proviruses, including the complete *gag, pol* and *env* genes, koalas from SA preferentially express the 5′ portion of *gag* and the 3′ portion of *env*, whereas *pol* transcripts are weak or undetectable. This may reflect ongoing endogenization events in which short variants of Gag and Env are preserved that mediate restriction to exogenous KoRV. Of note, the Fv1 restriction factor in mice is also not a full-length Gag protein but covers the first three-fourth of the retroviral Gag protein it is likely derived from (Bénit et al., 1997). Another explanation for why koalas from Queensland are not protected from KoRV is that expression of the full-length KoRV genes tolerizes the animals to the virus *in utero*, rendering their immune systems unable to recognize and respond to the exogenous virus. This may explain why many animals from Queensland do not have antibodies to KoRV and also do not elicit them upon vaccination (Fiebig et al., 2015; Waugh et al., 2016).

## HIV *En Route* to Endogenization?

Is HIV currently ongoing endogenization in humans? HIV is a complex retrovirus of the *lentivirus* genus. In contrast, ERVs are typically derived from simple retroviruses. The first endogenous lentivirus, RELIK (rabbit endogenous lentivirus-K) was identified only in 2007 (Katzourakis et al., 2007) [simple ERVs have been known since the 1960s (Weiss, 2006)], followed by the reports of several endogenous lentiviruses in primate (Gifford et al., 2008; Gilbert et al., 2009; Han and Worobey, 2015), ferret (Cui and Holmes, 2012), and weasel genomes (Han and Worobey, 2012). Still, the vast majority of known ERVs are derived from simple retroviruses, suggesting that endogenization of lentiviruses has been a relatively rare event, perhaps due to the recent evolutionary origin of lentiviruses (Katzourakis et al., 2007).

A prerequisite for endogenization to occur is the capability of a retrovirus to infect germline cells. Whether HIV can infect human germline cells is controversial. HIV-1 particles have been detected on the cell membrane and inside *in vitro* infected spermatozoa (Baccetti et al., 1994, 1998; Barboza et al., 2004; Cardona-Maya et al., 2009). In addition, HIV-1 proviral DNA has been detected by PCR in spermatozoa of HIV-1 infected individuals (Bagasra et al., 1994; Nuovo et al., 1994; Cardona-Maya et al., 2009). HIV-1 particles can associate with spermatozoa by binding to mannose receptor, which can transfer the virus into oocytes (Baccetti et al., 1994; Cardona-Maya et al., 2011). These findings indicate that sperm cells can be infected and subject to provirus integration, and can also act as vectors of HIV-1 virions to infect oocytes. Therefore, endogenization of HIV-1 appears to be possible in theory. Vertical transmission of proviral DNA through the germline, however, has not been demonstrated yet.

A recently reported HIV-1 patient controlling the infection without antiretroviral therapy despite not having the CCR5-Δ32 mutation or a protective HLA genotype suggests that the presence of HIV-1 proviruses in lymphocytes may protect against AIDS (Colson et al., 2014). PBMCs from this patient harbored defective HIV-1 proviruses and could not be superinfected with the same strain of HIV-1 *in vitro*, suggesting that the proviruses rendered the PBMCs resistant to infection. The proviruses harbored a number of premature stop codons likely introduced by the APOBEC3G restriction factor, however, some ORF were intact. The presence of apparently protective HIV-1 proviruses suggests that SIEx mediated by HIV-1 proviruses is likely possible. Thus, a potential germline infection with HIV-1 may confer inheritable resistance against HIV-1 induced disease.

## Protection Against Viral Infection by Endogenized Non-Retroviral Genes in Eukaryotes

Mammalian genomes not only contain ERVs and TEs, but also a number of sequences derived from *Bornaviridae, Filoviridae, Parvoviridae, Circoviridae, Rhabdoviridae*, and others (Belyi et al., 2010a,b; Horie et al., 2010; Katzourakis and Gifford, 2010; Aswad and Katzourakis, 2014). Genomic sequences from RNA viruses without an RT are likely processed pseudogenes originating from illegitimate reverse transcription and integration by the replication machinery of long interspersed nuclear elements (LINEs), or they have arisen from recombination with ERV RNA (Suzuki et al., 2014).

The best studied example of the function of non-retroviral endogenous virus sequences are Borna disease viruses (BDVs). BDVs are neurotropic negative sense ssRNA viruses causing fatal encephalitis (Borna disease) in horses, sheep and cattle (Carbone, 2001; Belyi et al., 2010b). These highly susceptible species have no detectable endogenous BDV sequences in their genomes (Belyi et al., 2010b). BDV also persistently infects other species, from avian to primate, and in experimental animals such as mice can induce behavioral changes without obvious signs of encephalitis. Interestingly, the genomes of primates, rats, and mice and squirrels, which are relatively resistant to the virus, harbor BDV sequences (Belyi et al., 2010b). This suggests a protective role of endogenous BDV sequences in protecting against disease caused by exogenous BDV.

Experimental evidence for protection mediated by endogenous BDV sequences has been obtained in squirrels. The genome of the 13-lined ground squirrel *Ictidomys tridecemlineatus* contains an endogenous bornavirus-like nucleoprotein (itEBLN) sequence that shares 77% amino acid similarity with current infectious BDV (Fujino et al., 2014). itEBLN colocalizes with the viral factory of BDV in the nucleus and suppresses viral replication and cell-to-cell spread *in vitro*, likely acting as a dominant negative nucleoprotein that is incorporated into BDV virions, which renders them non-infectious. Thus, itEBLN may serve as immune memory against exogenous BDV.

Like squirrels, humans usually do not experience Borna disease, with only three cases of fatal BDV-induced viral encephalitis reported to date resulting from zoonotic infections from squirrels (Hoffmann et al., 2015) and three more cases (two of which fatal) of human-to-human transmission during organ transplantation (Friedrich-Loeffler-Institut, 2018). All of the seven human endogenous bornavirus-like nucleoprotein elements (hsEBLN-1 through hsEBLN-7) are expressed as RNA in one or more tissues (Sofuku et al., 2015). At least one of them, hsEBLN-2, is also expressed as protein in human cells (Ewing et al., 2007). In primates and rodents, EBLNs are significantly enriched in piRNA clusters (Parrish et al., 2015). Three of the seven hsEBLN genes and three of five rodent EBLNs are located in piRNA clusters. Interestingly, piRNA cluster-located EBLNs in both rodents and primates produce bona fide piRNAs, which are antisense relative to the BDV nucleoprotein mRNA and are expressed in the testes. Whether piRNA-mediated inhibition of BDV infection occurs in germline cells, however, remains to be determined. As piRNAs can also be expressed in somatic cells including neurons (Lee et al., 2011), EBLN sequences may also protect from BDV infection in the brain, which may at least partially explain the resistance of species with endogenous EBLN sequences to viral encephalitis. Another potential mechanism by which EBLNs may protect from Borna disease is the induction of immune tolerance by *in utero* expression of EBLN protein (Horie, 2017). Tolerization of the adaptive immune system to the EBLN protein *in utero* may limit the immune response to the nucleoprotein during BDV infection. The BDV nucleoprotein is known as a major target for cytotoxic T cell responses (Planz and Stitz, 1999). *In utero* tolerization to this antigen may be protective, as most of the symptoms of fatal BDV infection arise from immune-mediated inflammation. A further antiviral mechanism might occur at the RNA level. EBLN RNAs could act as antisense transcripts to the genomic minus sense ssRNA genome of BDV that replicates in the nucleus (Horie, 2017). Aside from squirrels, primates and rodents, EBLN sequences have been identified in Afrotherians, bats, whales, birds, and lamprey (Kobayashi et al., 2016; Hyndman et al., 2018). It is conceivable that some of these elements also exert anti-viral functions.

The genomes of *Aedes* mosquitoes which are important vectors for human pathogenic flaviviruses such as Dengue and Zika contain various endogenous flaviviral sequences (Suzuki et al., 2017). piRNAs and siRNAs are produced from these endogenous viruses and might play a role in antiviral defense. It is known that small RNAs play an important role in antiviral defense in insects (Cullen et al., 2013).

A variation of SIEx can also be mediated by viruses that infect other viruses, termed virophages. The protozoan *Cafeteria roenbergensis* is infected by *Cafeteria roenbergensis* virus (CroV), a giant virus that causes lysis of the host (Fischer and Suttle, 2011). CroV is infected by the virophage Mavirus (Fischer and Hackl, 2016). *C. roenbergensis* cells co-infected with CroV and Mavirus are protected from lysis, as Mavirus inhibits replication of CroV. Interestingly, Mavirus can integrate into the genome of *C. roenbergensis* where it stays inactive until the cell is infected with CroV. The activated Mavirus then inhibits CroV replication, thus providing an adaptive, inducible immunity of *C. roenbergensis* against detrimental CroV infection. Stably integrated into the *C. roenbergensis* genome, Mavirus is passed on to the next generation of the protozoan, which can be regarded as a simple form of an inheritable immune system.

Polintons, TEs related to virophages, are found in the genomes of diverse eukaryotic species and likely originate from viruses with an exogenous form (Koonin and Krupovic, 2018). They may represent endogenized virophages that, unlike Mavirus in *C. roenbergensis*, have lost the ability to form virions. Polintons may have been recruited by their eukaryotic hosts as a defense against past or present viruses, whose identity remains to be determined.

## Adaptive Immunity of Jawed Vertebrates: V(D)J Recombination

In contrast to the adaptive immune system of prokaryotes, CRISPR-Cas, immunological memory in vertebrates is restricted to somatic cells and is therefore not inherited to the next generation. In jawed vertebrates, the diversity of immunoglobulins/antibodies and TCRs is generated by V(D)J recombination, in which variable (V), diversity (D) and joining (J) segments are recombined. Further antibody diversification is then achieved by somatic hypermutation (Kapitonov and Koonin, 2015).

The ability to produce diversity of antibodies and TCRs in jawed vertebrates developed at around 500 mya (Kapitonov and Koonin, 2015). Both the Rag1 and Rag2 proteins required for V(D)J recombination are found in one genomic locus and originate from a *Transib* transposon that today is found in the genomes of starfish, oysters and sea urchins, but not anymore in those of jawed vertebrates (Kapitonov and Koonin, 2015). Rag1 is the nuclease responsible for V(D)J recombination, which contains an RNase H-like domain with the conserved DDE catalytic triad.

## Possible Evolution of Components of RNA Interference From Viruses

**FIGURE 4:**
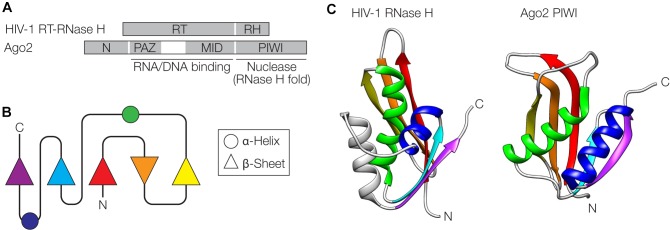
Similarities between retroviral reverse-transcriptase (RT)-RNase H and Argonaute. **(A)** Domain organization of the HIV-1 RT-RNase H and Argonaute 2 (Ago2) from *Pyrococcus furiosus*. The RT domain of RT-RNase H and the Piwi Argonaute Zwille (PAZ) and middle (MID) domains of Ago2 contain regions involved in nucleic acid binding. The RNase H (RH) and PIWI domains adopt an RNase H fold and serve as nucleases. **(B)** Schematic of the RNase H fold. **(C)** The RNase H and PIWI domains are shown next to each other, with α-helices and β-sheets color-coded as in panel **(B)**. PDB accession numbers are 2I5J for the RNase H (Himmel et al., 2006) and 1U04 for Ago2 (Song et al., 2004) The gray helix in the HIV structure is an additional helix not shown in **(B)**. Structures were visualized with UCSF Chimera (Pettersen et al., 2004).

The retroviral replication machinery and Argonaute (Ago)-mediated interference pathways against invading nucleic acids share some surprising similarities at the structural and functional level (Moelling et al., 2006a, 2017; Moelling and Broecker, 2015). At the core of retroviral replication is the reverse transcriptase-RNase H (RT-RNase H) that resembles Ago proteins consisting of PAZ (N-terminal), MID (central) and PIWI (C-terminal) domains (Figure 4A). The C-terminal domains of both proteins adopt an RNase H-fold, one of the most ancient and abundant protein folds found in nature (Figure 4B,C) (Wang et al., 2006; Ma et al., 2008; Majorek et al., 2014). These domains have nuclease activity and cleave the target RNA/DNA (the template retroviral RNA during retroviral replication or the nucleic acid targeted by Ago *via* the guide nucleic acid) (Mölling et al., 1971; Song et al., 2004). The N-terminal domains of both proteins, RT of the RT-RNase H and PAZ of Ago, among other functions, serve as nucleic acid binding modules that direct the cleavage specificity of the RNase H domains. The RNA/DNA binding activity in the RT domain of RT-RNase H is located in conserved residues binding the template RNA strand (“template grip”) as well as the opposite cDNA strand (“primer grip”) (Dash et al., 2008). In the case of Ago, the PAZ domain is an oligonucleotide-binding domain that interacts with the 3′ end of the guide (Song et al., 2004). Further interactions with the 5′ end of the guide are made by the MID domain (Lima et al., 2009). In both RT-RNase H and Ago, the N-terminal domains fused to the RNase H domain determine the specificity of the nuclease activity. The widespread presence of Ago proteins in prokaryotes and eukaryotes with conserved structures and functions suggests an ancient evolutionary origin, possibly before the last eukaryotic common ancestor (Swarts et al., 2014). The diverse Ago proteins can act as RNA- or DNA-guided nucleases and can cleave RNA or DNA through the RNase H-like PIWI domain. In both prokaryotes and eukaryotes, Ago-centered defense can be regarded as a mechanism of innate immunity (Koonin and Krupovic, 2015).

RNAi is triggered by siRNAs, short (21–24 nucleotides) double-stranded RNAs with two nucleotide overhangs at the 3′ ends, which are produced by Dicer from longer dsRNA molecules. A DNA molecule structurally related to siRNAs, a partially double-stranded hairpin-loop 54-mer oligodeoxynucleotide (ODN) is an efficient inducer of the HIV-1 RT-RNase H, leading to cleavage of the retroviral RNA genome (Moelling et al., 2006b). Interestingly, the human AGO2 protein can use both siRNAs and ODNs to find and cleave target RNA in a sequence-specific manner *in vitro* – albeit with lower efficiency with the ODN (Moelling et al., 2006a). *Vice versa*, siRNAs are recognized by the HIV-1 RT-RNase H to induce target RNA cleavage but with lower efficiency than the corresponding ODN. These common activities suggest an evolutionary relationship between the RT-RNase H and AGO2. Another component required for RNAi, the RNA-dependent RNA polymerase (RdRP) that amplifies siRNAs, likely originated from a phage (Shabalina and Koonin, 2008). The RdRP was likely present in the last eukaryotic common ancestor and is still active in plants and nematodes (Shabalina and Koonin, 2008).

While eukaryotic Ago proteins are generally believed to only use RNA as guides, prokaryotic Ago proteins have been demonstrated previously to also accept DNA as guides, with important functions *in vivo* (Yuan et al., 2005). The abovementioned activity of AGO2 with a DNA guide against HIV-1 RNA in the test tube suggests that mammalian RNAi may also be triggered by DNA guides to serve biological functions. Indeed, DNA molecules have been shown to bind to the PAZ domain of AGO2 and localize into mRNA-degrading P bodies, hallmark features of RNAi-mediated degradation (Castanotto et al., 2015). It has been suggested that cytosolic genomic DNA (cgDNA) functions as natural antisense mechanism triggering RNA degradation, perhaps involving AGO2 (Asada et al., 2018). Single-stranded cgDNA of TE origin can be detected in mammalian cell lines and may act as a natural antisense mechanism against the RNA of retrotransposons, especially ERVs (Stetson et al., 2008; Asada et al., 2018). However, under normal conditions *in vivo* the exonuclease TREX1 appears to degrade single-stranded cgDNA, preventing the antisense inhibition. Notably, loss of function mutations in the human *TREX1* gene cause Aicardi-Goutières Syndrome (Crow et al., 2006), an autoimmune disease characterized by the accumulation of cytosolic ssDNA (Yang et al., 2007), including ssDNA of TEs, especially of ERV origin (Stetson et al., 2008). Thus, cgDNA may restrict TE transcripts *via* RNAi under conditions without or with low TREX1 expression.

Another mechanism by which TE-derived nucleic acids lead to inhibition of TE expression is the endogenous siRNA (endosiRNA) pathway (Ghildiyal et al., 2008; Nandi et al., 2016; Berrens et al., 2017). In this pathway, TE sense-antisense RNA pairs that arise, for instance, from intron-located TEs (Figure 1) are subject to RNAi, involving Dicer and AGO2, which suppresses TE activity in the mammalian germline (Berrens et al., 2017), the mammalian brain (Nandi et al., 2016) as well in somatic cells of *Drosophila* (Ghildiyal et al., 2008). It appears possible that a similar siRNA-based mechanism may also act against exogenous retroviral RNA if there is sufficient complementarity between an antisense-transcribed ERV and the mRNA of the exogenous retrovirus (Figure 1). Indeed, mammalian RNAi has been demonstrated *in vitro* to also act against exogenous viruses. This includes RNAi-mediated restriction of enteroviruses (Qiu et al., 2017), encephalomyocarditis virus and Nodamura virus (Maillard et al., 2013), Influenza virus (Matskevich and Moelling, 2007; Li et al., 2016), reovirus and Sindbis virus (Maillard et al., 2016). The biological relevance of siRNA in mammalian cells, however, is subject to debate, as many mammalian viruses efficiently counteract RNAi (tenOever, 2017; Tsai et al., 2018). Moreover, mammalian antiviral RNAi is usually only detected in cells defective in IFN signaling and may be restricted to embryonic stem cells. In plants, nematodes and invertebrates, however, RNAi plays an important role in antiviral defense (Cullen et al., 2013).

## Conclusion

Recruitment of sequences from viruses, TEs, and other MGEs for immune defense mechanisms in prokaryotes and eukaryotes is strikingly common. Nucleases are involved in many immune systems, either to cleave invading DNA or to mediate genome editing events (Figure 1). Many of these are RNase H-like nucleases, including Ago/Piwi proteins involved in foreign nucleic acid cleavage in prokaryotes and in RNAi in eukaryotes, some CRISPR-Cas effector nucleases (Cas9 and Cas12), and the Rag1 protein that mediates V(D)J recombination. Thus, RNase H-like molecules are involved in different prokaryotic and eukaryotic immune systems of various origin. The fact that genomes of almost all cellular organisms harbor large numbers of MGEs suggests that yet unknown functionalities may be identified in the future. The recent discovery that defense islands in bacterial genomes are enriched with sequences of TEs further pinpoints their important role in immune defense mechanisms in prokaryotes. Their potential functions remain to be elucidated. In eukaryotes whose genomes usually contain even more TE sequences than prokaryotic ones, additional immune functions are also expected to be discovered. This includes TE-derived conserved genes such as *HARBI1*, which is conserved across vertebrates and originated from a Harbinger transposase, with yet unknown functions (Koonin and Krupovic, 2015).

It has to be noted that antiviral defense is by far not the only function of endogenized viruses and TEs. For example, deletion of all replication-deficient prophages in *E. coli* has resulted in fitness deficits under diverse environmental conditions, including increased susceptibility to antibiotics and osmotic stress, slower cell growth and reduced biofilm formation (Wang et al., 2010). In eukaryotes, TEs and ERVs do not only modulate IFN response genes and constitute antiviral defense mechanisms, but also play distinct roles in cell differentiation, stem cell pluripotency and embryonic development, amongst others (Chuong et al., 2017), and the industrial melanism mutation of peppered moths has been shown to be caused by a TE insertion (Van’t Hof et al., 2016). These examples highlight the multifaceted roles of TEs and viral sequences in pro- and eukaryotes. However, given their diverse roles in various immune systems (Figure 1), it appears that recruitment of TEs, viral sequences and other MGEs for antiviral defense mechanisms has been a major driving force in the evolution of cellular life.

## Author Contributions

Both authors have written the manuscript and approved its final version.

## Conflict of Interest Statement

The authors declare that the research was conducted in the absence of any commercial or financial relationships that could be construed as a potential conflict of interest.
